# Proinflammatory and Hepatic Features Related to Morbidity and Fatal Outcomes in COVID-19 Patients

**DOI:** 10.3390/jcm10143112

**Published:** 2021-07-15

**Authors:** Omar Ramos-Lopez, Rodrigo San-Cristobal, Diego Martinez-Urbistondo, Víctor Micó, Gonzalo Colmenarejo, Paula Villares-Fernandez, Lidia Daimiel, J. Alfredo Martinez

**Affiliations:** 1 Medicine and Psychology School, Autonomous University of Baja California, Tijuana 22390, Mexico; oscar.omar.ramos.lopez@uabc.edu.mx; 2 Precision Nutrition and Cardiometabolic Health, IMDEA Food Institute, CEI UAM+CSIC, 28049 Madrid, Spain; rodrigo.sancristobal@imdea.org; 3 Hospital Universitario HM Sanchinarro, 28050 Madrid, Spain; dmurbistondo@gmail.com (D.M.-U.); pvillares@hmhospitales.com (P.V.-F.); 4 Nutritional Control of the Epigenome Group, IMDEA Food Institute, CEI UAM+CSIC, 28049 Madrid, Spain; victor.mico@imdea.org (V.M.); lidia.daimiel@imdea.org (L.D.); 5 Biostatistics and Bioinformatics Unit, IMDEA Food Institute, CEI UAM+CSIC, 28049 Madrid, Spain; gonzalo.colmenarejo@imdea.org; 6 Department of Nutrition, Food Science, Physiology and Toxicology, Centre for Nutrition Research, University of Navarra, 31009 Pamplona, Spain; 7 Navarra Institute for Health Research (IdiSNA), 31009 Pamplona, Spain; 8 Spanish Biomedical Research Centre in Physiopathology of Obesity and Nutrition (CIBERobn), 28029 Madrid, Spain

**Keywords:** liver markers, inflammation, morbidity, mortality, personalized medicine

## Abstract

Objective: to screen putative associations between liver markers and proinflammatory-related features concerning infectious morbidity and fatal outcomes in COVID-19 patients. Methods: a total of 2094 COVID-19 positive patients from the COVID-DATA-SAFE-LIFES cohort (HM hospitals consortium) were classified according to median values of hepatic, inflammatory, and clinical indicators. Logistic regression models were fitted and ROC cures were generated to explain disease severity and mortality. Results: intensive care unit (ICU) assistance plus death outcomes were associated with liver dysfunction, hyperinflammation, respiratory insufficiency, and higher associated comorbidities. Four models including age, sex, neutrophils, D-dimer, oxygen saturation lower than 92%, C-reactive protein (CRP), Charlson Comorbidity Index (CCI), FIB-4 and interactions with CRP, neutrophils, and CCI explained ICU plus death variance in more than 28%. The predictive values of ROC curves were: FIB-4 (0.7339), AST/ALT ratio (0.7107), CRP (0.7003), CCI index (0.6778), neutrophils (0.6772), and platelets (0.5618) concerning ICU plus death outcomes. Conclusions: the results of this research revealed that liver and proinflammatory features are important determinants of COVID-19 morbidity and fatal outcomes, which could improve the current understanding of the COVID-19 physiopathology as well as to facilitate the clinical management and therapy decision-making of this disease under a personalized medicine scope.

## 1. Introduction

Coronavirus disease 2019 (COVID-19), caused by the Severe Acute Respiratory Syndrome Coronavirus 2 (SARS-COV-2), has being declared as a pandemic by the World Health Organization (WHO) in March 2020 based on the rises in the daily number of new cases, fast and ample spread, lethality, and the lack of effective antiviral treatments [1]. Since COVID-19 emergence in Wuhan, China, in December 2019, millions of COVID-19 cases have been reported worldwide, with a wide spectrum of respiratory presentations and multisystemic complications [2].

The excessive immunological reaction to the virus (known as “cytokine storm”) by the host is largely responsible for the respiratory manifestations of COVID-19, encompassing pneumonia and acute respiratory distress syndrome (ARDS); however, in some patients this response may also involve hepatic, gastrointestinal, cardiac, renal, neurological, and hematological affectations [3]. Concerning liver injuries, large-scale case studies indicate that up to 11% of patients developed liver comorbidities, and more than 50% of cases reported abnormal levels of transaminases during disease progression, whereas liver dysfunction was more prevalent in severe COVID-19 patients [4]. In such patients, liver damage seems to be directly caused by the viral infection of liver cells, drug toxicity, and immune-mediated inflammation [5]. However, further studies are needed to understand and elucidate the precise causes of liver disease in COVID-19.

Until now, certain clinical, demographic, and phenotypical factors have been reported to be associated with the evolution and severity of COVID-19, encompassing age, sex, ethnicity, underlying medical conditions such as obesity, diabetes, and hypertension, poverty and crowding, pregnancy, and the use of certain medications and genetics [6,7]. Others include elevated levels of proinflammatory cytokines, liver enzymes, coagulation factors, body temperature, and unhealthy lifestyle such as smoking and alcoholic drinks consumption [8]. Nonetheless, there is a constant need for the search for easily accessible, rapid and accurate markers related to the course of COVID-19, which could contribute to improving the individualized clinical management and monitoring of the progression of this infection through an integrative precision medicine approach [9]. The aim of this research was to screen putative associations between available liver markers and proinflammatory-related features concerning infectious morbidity and fatal outcomes in COVID-19 patients.

## 2. Methods

### 2.1. Database and Study Variables

In this retrospective study, data from emergency admission of 2094 COVID-19 positive patients from the COVID-DATA-SAFE-LIFES cohort were analyzed. This cohort contains data on 2226 patients treated for COVID-19 in the HM group hospitals in the first wave of infections (March–May 2020), which has been made available to the international scientific community for study upon appropriate request and approval by a Committee expressly appointed by the hospital consortium (CEIm HM Hospitales Ref No. 20.05.1627-GHM) and under appropriate ethical protocols (Helsinki Declaration).

All data were recorded according to in-hospital protocols, which were harmonized and curated for further analysis in the R software (version 4.0.3). The study variables analyzed in this investigation at baseline comprised age, sex, oxygen saturation, leukocytes, lymphocytes, neutrophils, platelets, basophils, eosinophils, monocytes, C-reactive protein (CRP), D-dimer, fibrinogen, ferritin, procalcitonin, glucose, cholesterol, lactate dehydrogenase (LDH), gamma glutamyl transferase (GGT), aspartate aminotransferase or glutamic oxaloacetic transaminase (AST/GOT), and alanine transaminase or glutamate pyruvate transaminase (ALT/GPT). The following inflammatory-related ratios were calculated: international normalized ratio (INR), AST/ALT ratio (AAR), basophil-to-lymphocyte ratio (BLR), neutrophil-to-lymphocyte ratio (NLR), platelet-to-lymphocyte ratio (PLR), eosinophil-to-basophil ratio (EBR), eosinophil-to-lymphocyte ratio (ELR), and lymphocyte-to-monocyte ratio (LMR). Moreover, the Charlson Comorbidity Index (CCI) was computed to express the sum of co-morbidities. As non-invasive methods for predicting liver fibrosis [10], the following scores were calculated: 

AST to Platelet Ratio Index (APRI): APRI = [(AST/upper limit of the normal AST range) × 100]/Platelet Count.

Fibrosis-4 index (FIB-4): FIB-4 = Age (years) × AST (U/L)/[platelet count(10^9^/L) × ALT^1/2^ (U/L)].

### 2.2. Statistical Analyses

Quantitative and qualitative variables were expressed as means ± standard deviations (SD) and as number and percentage, respectively. Chi-square and Student’s t-test were applied to analyze differences between qualitative and quantitative variables, as appropriate. Death and ICU were combined and used as main outcomes since these are objective criteria of poor prognosis, as reported elsewhere [11]. Phenotypical and metabolic characteristics of the COVID-19 patients were compared by the median values of hepatic (FIB-4), inflammatory (CRP, neutrophils), and clinical markers (CCI index and oxygen saturation) by Student’s *t*-test. Multivariable logistic regression models were fitted to explain disease severity and mortality, with age and sex as covariates. Age was excluded from the CCI index in the models to avoid colinearity. Statistical associations were calculated by univariate logistic regression tests. In addition, area under the receiver operating characteristic (ROC) curves were built to evaluate the predictive values of clinically relevant variables. Statistical analyses were performed in the statistical program Stata 12 (StataCorp LLC, College Station, TX, USA; www.stata.com (accessed on 2 May 2021)) and IBM SPSS 20 (IBM Inc., Armonk, NY, USA). Statistical significance was set at *p* value lower than 0.05, with bilateral test.

## 3. Results

The clinical and phenotypical characteristics of COVID-19 patients based on respiratory insufficiency, comorbidity, or need of intensive care plus mortality risk are reported (Table 1). On average, individuals with oxygen saturation lower than 92%, CCI index equal or higher than 3, and those who underwent ICU or who died were male, older and presented higher levels of leukocytes, neutrophils, CRP, D-dimer, LDH, FIB-4 as well as elevated ratios of AAR, basophil-to-lymphocyte, neutrophil-to-lymphocyte, platelet-to-lymphocyte, and lymphocyte-to-monocyte than their counterparts. Conversely, no differences between groups were observed for basophils, procalcitonin, glucose, cholesterol, and GPT measurements.

Similar features were found when compared the median values of inflammatory (CRP and neutrophils) and liver (FIB-4) markers in COVID-19 patients (Table 2).

Logistic regression models using relevant biochemical and clinical variables to predict ICU plus death outcome were constructed, with age and sex as covariates. Interestingly, four models were statistically significant (*p* < 0.001) and explained ICU plus death variance in more than 28% (Table 3a–d). The models included age, sex, neutrophils, D-dimer, oxygen saturation < 92%, CRP, CCI index, FIB-4, and the following interactions: CCI index × CRP (Table 3a); FIB-4 * CCI index (Table 3b); FIB-4 * CRP (Table 3c); and FIB-4 * neutrophils (Table 3d), respectively. The four interactions were statistically significant in the corresponding models. 

**Figure 1:**
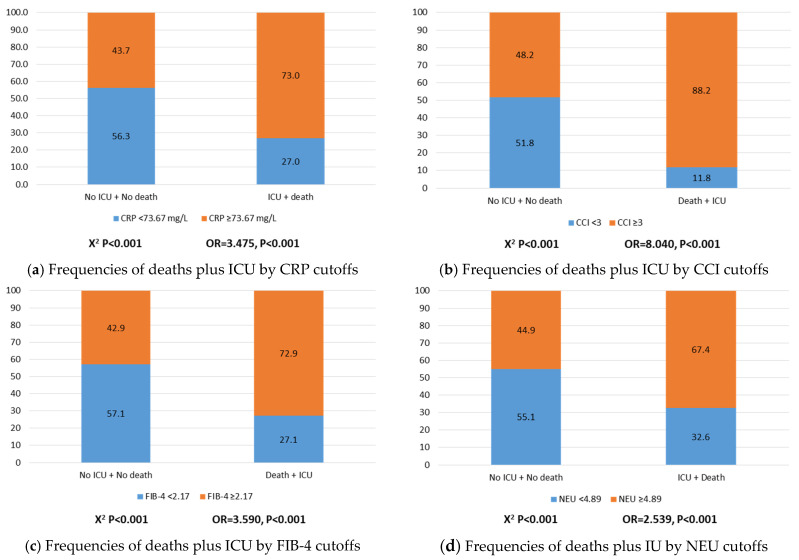

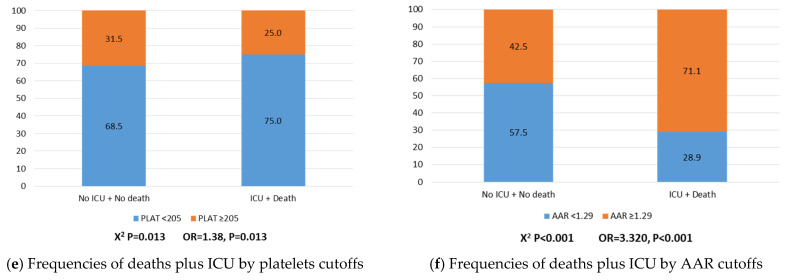
Frequencies and odds ratios (OR) of ICU plus death by the cutoffs (median) values of CRP, CCI index, FIB-4, neutrophils, platelets, and AAR ratio.

The empirical frequencies and odds ratios (OR) of ICU plus death by the cutoffs (median) values of CRP, CCI index, FIB-4, neutrophils, platelets, and AAR ratio are depicted (Figure 1a–f). Compared to patients who did not enter to ICU and did not die, higher risks of ICU plus death were found when CRP levels were equal or higher than 73.67 mg/L (OR = 3.475, *p* < 0.001, Figure 1a); CCI index equal or higher than 3 (OR = 8.040, *p* < 0.001, Figure 1b); FIB-4 score equal or higher than 2.17 (OR = 3.590, *p* < 0.001, Figure 1c); neutrophils equal or higher than 4.89 × 10^9^/L (OR = 2.539, *p* < 0.001, Figure 1d); and AAR ratio equal or higher than 1.29 (OR = 3.320, *p* < 0.001, Figure 1f). Instead, platelets equal or higher than 205 × 10^9^/L protected for ICU pus death (OR = 0.723, *p* = 0.013, Figure 1e).

**Figure 2:**
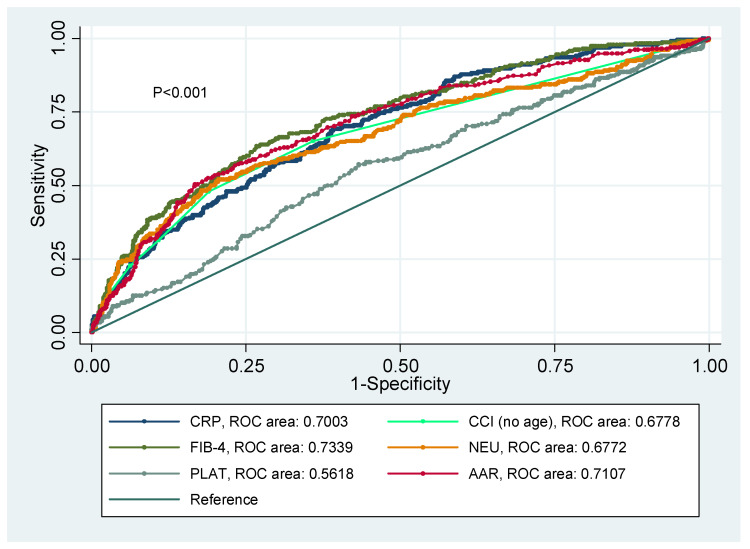
ROC curves showing the predictive value of CCI (no age), CRP, NEU, FIB-4, PLAT, and AAR concerning COVID-19 outcomes (ICU plus Death).

ROC curves were constructed to estimate and compare the predictive value of liver and proinflammatory markers concerning ICU plus death (Figure 2). The best predictor was FIB-4 (0.7339), followed by AAR (0.7107), CRP (0.7003), CCI index (0.6778), neutrophils (0.6772), and platelets (0.5618), all of them statistically significant (*p* < 0.001). 

## 4. Discussion

As a result of the increased availability of data and collaborations between researchers, efforts have been made for the evaluation of laboratory tests and other phenotypical information as biomarkers related to COVID-19 disease severity [9]. This study should be considered a proof of concept, where biochemical and clinical variables significantly explained morbid and fatal outcomes in COVID-19 patients, including neutrophils, CRP, oxygen saturation < 92%, FIB-4, D-dimer and CCI index, which evidence the involvement of predominately liver and proinflammatory features in the evolution of this disease. These findings may enable early categorization of infected patients based on the risk of death or intensive care assistance, thus facilitating a more precise clinical management as well as the optimization of health resources and medical personnel [7].

In agreement with our results, neutrophils have been highlighted as essential effector cells in COVID-19 physiopathology through the stimulation of a hyperinflammation state in the lungs by enhanced degranulation of primary granules and the secretion of proinflammatory cytokines as well as the induction of oxidative stress via reactive oxygen species release [12]. In this context, bioinformatic analyses revealed that neutrophil activation is one of the most stimulated biological processes in the SARS-CoV infection [13]. Moreover, it has been reported the association of NLR with critical illness in COVID-19 patients [14].

Likewise, some investigations have confirmed the utility of CRP as prognostic factor in COVID-19 since it serves as an early marker of infection, inflammation, and tissue damage [15]. For example, CRP levels were independent discriminators of severe/critical illness on admission and a good predictor of adverse outcome in COVID-19 patients [16]. In hospitalized patients, median CRP values (206 mg/L) were significantly higher in the patients who died compared to those who survived, and increased linearly during the first week of hospitalization, which supports the utility of daily CRP monitoring in risk prognostication [17]. Accordingly, it has been documented that the risk of developing severe events in COVID-19 patients is increased by about 5% for every one-unit increase in CRP levels [18]. Interestingly, elevated levels of CRP (76.51 mg/L) correlated with lower oxygen saturation (<90%), indicating a relationship of these markers and a complementary utility in the prognosis of COVID-19 disease [19]. Indeed, oxygen saturation levels below 92% significantly contributed to predict ICU plus death in this sample. This hallmark is in agreement with the criteria for diagnosis of COVID-19-associated pneumonia and disease severity [20], as postulated in the guidelines of the World Health Organization for the Clinical Management of COVID-19 (https://apps.who.int/iris/handle/10665/332196 (accessed on 2 May 2021)). Certainly, 92% is under the current target oxygen saturation range (92–96%) for patients with COVID-19 recommended by the National Institutes of Health (https://www.covid19treatmentguidelines.nih.gov/critical-care/oxygenation-and-ventilation/ (accessed on 2 May 2021)).

Another important finding of this study was the interplay of FIB-4 in COVID-19 disease severity by interacting with proinflammatory and comorbid features. Thus, two statistical interactions were found concerning FIB-4 and inflammatory markers, where a higher FIB-4 score combined with increased levels of neutrophils and CRP were associated with more instances of ICU plus death (data not shown). These results suggest that an elevated FIB-4 score exacerbates the progression of the inflammatory process, and also suggests an organ-specific influence of inflammation as a prognostic marker. Besides, a significant interaction between CCI and FIB-4 in relation to death plus ICU was found in this research (data not shown), which suggest that when FIB-4 is low, the CCI dominates the entry to ICU admission and the risk of death; however, when FIB-4 is high (above 20), a preservative effect is found. This finding may be explained by the fact that the set of comorbidities (measured by CCI) has a greater influence on the outcomes of patients with COVID-19 than only liver fibrosis (measured by FIB-4). FIB-4 is not only an accurate marker of liver fibrosis, but it is also related to coagulation and oxidative stress since it takes into account age and the serum levels of transaminases (ALT and AST) and platelets, all of which have been consistently identified as potential risk factors of severe cases with COVID-19 in a recent meta-analysis [21]. Furthermore, elevation of this FIB-4 (equal or higher than 2.67) was associated with poor clinical outcomes in middle-aged patients with COVID-19, including required mechanical ventilation and ICU admission [22]. Moreover, FIB-4 was also related with increased risk of mortality in hospitalized patients with COVID-19 as well as with lower survival [23,24]. In addition, FIB-4 positively correlated to SARS-CoV-2 viral load and the levels of inflammatory cytokines [25]. Besides FIB-4, AAR was another liver marker also associated (equal or higher than 1.29) with an increased risk of ICU plus death in this research. Similarly, a retrospective study reported that AAR higher than 1 highly correlated with liver injury in conjunction with other proinflammatory variables [26]. Despite more investigation in this fled is necessary, these results evidence the involvement of liver damage in the evolution of COVID-19 and highlight the importance of evaluate liver status in the clinical setting. Although the role of liver disease in COVID-19 remains unclear, it has been hypothesized that liver injury is associated with innate immune dysfunction, which could enhance susceptibility to an acute proinflammatory response (cytokine storm) leading to severe outcomes in patients with COVID-19 by exacerbating the hyperinflammatory state [27,28]. Of note, although the presence of previous liver disease might artifact our findings, the low prevalence in the population (only 53 patients with liver disease) might reduce the confounding effect of this issue. In fact, no significant differences in the performance of the statistical models were found when patients with liver disease were removed.

In relation to the association of abnormal coagulation parameters with poor outcomes in COVID-19 patients, a meta-analysis evidenced that patients with a composite clinical end point, defined as all-cause mortality, ICU admission or ARDS, had elevated levels of D-dimer (standard mean difference of 1.67 µg/mL) than their counterparts [29]. In fact, results from another meta-analysis of 13 cohort studies revealed that severe COVID-19 infection was related to D-dimer higher than 0.5 μg/mL on admission [30]. 

Regarding comorbidity, in this study, the CCI index was included in the predictive models of ICU plus death mainly as an adjustment variable. The CCI has been commonly used in clinical research as a correction factor in a set of prognostic models due to proven consistency, validity, and reliability as supported by the results of several studies. In COVID-19, multivariate regression analysis showed that CCI was a prognostic factor for COVID-19-related mortality in patients hospitalized for pneumonia [31]. Additionally, CCI score above 0 was associated with an increased risk of severe outcome and death after controlled for age and sex [32]. In a meta-analysis, a 16% higher risk of mortality was attributed by each per point increase of CCI score [33].

On the one hand, the strengths of this investigation include a large sample screened and the use of robust statistical approaches for data depuration and the comparative predictive analyses. In this context, on the most important findings of this research is the integration of different predictors of COVID-19 outcomes including liver and pro-inflammatory features as well as the screening of potential interactions among these factors, which suggest that the prognostic value of these markers depends upon the behavior of concomitant variables influencing COVID-19 disease and that there is a mutual influence concerning the result. On the other hand, the fact that the population analyzed in this study has mainly European ancestry, the findings of this study could not be applied to groups with other ethnicity and exposed to diverse environmental factors. For instance, in Latin America, variables such as the high rates of obesity, the adoption of hepatopathogenic diets, and a sedentary lifestyle could exacerbate liver damage and a hyperinflammatory state in COVID-19 [34]. Moreover, the exploration of other variables influencing liver health and the immune response including the gut microbiota, genetic background, epigenetic signatures, metabolomic profiles, and interactions with specific lifestyle factors could be part of the scenario [35]. Additionally, although hyperinflammation worse COVID-19 infection, caution must be taken concerning the interpretation of the results since there can be wide fluctuations in levels of inflammatory markers during the time frame from admission to collection of labs. 

In conclusion, the results of this research suggest that liver and proinflammatory features are important determinants of COVID-19 morbid and fatal outcomes. This information could contribute to improve the current comprehension of the COVID-19 physiopathology and the clinical management and therapy decision-making of this disease under a precision medicine approach [36]. Current results evidence that the hepatic responses may have a role in prognosis, treatment, and understanding of COVID-19.

## Figures and Tables

**Table 1:** Clinical and phenotypical characteristics of COVID-19 patients based on respiratory insufficiency (oxygen saturation), comorbidities (CCI), and ICU plus death outcomes.

	Oxygen Saturation (SO_2_%)			CCI			ICU and Death		
Variable	≥92% (*n* = 494)	<92% (*n* = 1314)	*p*	<3 (*n* = 781)	≥3 (*n* = 994)	*p*	No ICU + No Death (*n* = 1645)	ICU + Death (*n* = 449)	*p*
Age	66.7 ± 16.7	72.8 ± 13.4	**<0.001**	52.2 ± 11.7	76.3 ± 10.7	**<0.001**	64.4 ± 16.0	76.2 ± 14.1	**<0.001**
Sex (F/M)	548/766	176/318	**0.019**	281/500	428/566	**0.003**	677/968	148/301	**0.002**
CCI	2.90 ± 2.38	4.01 ± 2.41	**<0.001**	1.00 ± 0.83	4.87 ± 1.93	**<0.001**	2.70 ± 2.21	5.15 ± 2.55	**<0.001**
Oxygen saturation (SO_2_%)	95.4 ± 2.0	84.4 ± 9.1	**<0.001**	94.0 ± 4.3	91.0 ± 8.3	**<0.001**	93.6 ± 5.1	87.2 ± 10.8	**<0.001**
Leukocytes (×10^9^/L)	7.13 ± 3.77	9.09 ± 6.04	**<0.001**	6.89 ± 3.15	8.00 ± 5.32	**<0.001**	7.14 ± 3.63	9.62 ± 6.65	**<0.001**
Lymphocytes (×10^9^/L)	1.27 ± 1.51	1.01 ± 0.79	**<0.001**	1.23 ± 0.60	1.15 ± 1.61	0.172	1.24 ± 1.30	1.04 ± 1.27	**0.007**
Neutrophils (×10^9^/L)	5.22 ± 3.13	7.37 ± 4.55	**<0.001**	5.08 ± 2.98	6.16 ± 4.10	**<0.001**	5.30 ± 3.20	7.79 ± 4.90	**<0.001**
Platelets (×10^9^/L)	219.4 ± 93.5	231.2 ± 98.4	**0.023**	225.6 ± 91.4	218.1 ± 94.9	0.107	227.4 ± 95.7	210.6 ± 100.1	**0.004**
Basophils (×10^9^/L)	0.022 ± 0.021	0.022 ± 0.021	0.526	0.021 ± 0.022	0.022 ± 0.020	0.475	0.021 ± 0.020	0.022 ± 0.021	0.400
Eosinophils (×10^9^/L)	0.05 ± 0.26	0.02 ± 0.06	**0.032**	0.04 ± 0.08	0.04 ± 0.29	0.527	0.05 ± 0.23	0.02 ± 0.05	**0.036**
Monocytes (×10^9^/L)	0.56 ± 0.37	0.68 ± 3.30	0.254	0.52 ± 0.29	0.63 ± 2.44	0.241	0.54 ± 0.30	0.75 ± 3.67	**0.044**
CRP (mg/L)	79.3 ± 83.2	158.4 ± 113.8	**<0.001**	83.4 ± 85.0	114.7 ± 107.5	**<0.001**	87.3 ± 87.1	161.3 ± 124.8	**<0.001**
D-dimer (μg/mL)	1.97 ± 6.72	3.33 ± 8.80	**0.003**	1.08 ± 2.56	3.19 ± 10.04	**<0.001**	1.75 ± 5.5	4.92 ± 13.21	**<0.001**
Fibrinogen (mg/dL)	638.7 ± 175.3	772.9 ± 348.4	**0.035**	696.1 ± 209.6	683.4 ± 193.8	0.763	646.8 ± 176.3	776.4 ± 307.0	**0.002**
Ferritin (ng/mL)	867 ± 701	2870 ± 2931	**<0.001**	2030 ± 3427	1313 ± 1345	0.226	1363 ± 2213	1812 ± 1484	0.392
Procalcitonin (ng/mL)	0.08 ± 0.03	1.02 ± 2.01	0.157	0.33 ± 0.89	1.00 ± 1.76	0.142	0.41 ± 0.89	0.89 ± 1.64	0.209
Glucose (mg/dL)	208.0 ± 97.6	177.0 ± 30.7	0.545	128.5 ± 34.1	216.8 ± 102.5	0.117	200.2 ± 105.7	169.0 ± 60.0	0.545
Cholesterol (mg/dL)	142.7 ± 31.5	144.5 ± 2.1	0.943	142.0 ± 4.2	142.8 ± 25.7	0.971	145.0 ± 39.6	133.1 ± 31.1	0.734
LDH (U/L)	546.1 ± 257.7	786.6 ± 411.2	**<0.001**	562.3 ± 256.0	646.9 ± 406.9	**<0.001**	563.5 ± 249.3	816.1 ± 570.5	**<0.001**
GGT (U/L)	74.2 ± 91.4	157.0 ± 345.4	**0.025**	74.5 ± 93.3	74.0 ± 170.0	0.978	74.4 ± 90.1	89.2 ± 211.7	0.420
GOT (U/L)	43.1 ± 110.9	58.7 ± 90.9	**0.012**	44.3 ± 32.9	50.9 ± 141.6	0.265	42.4 ± 32.8	66.6 ± 208.2	**<0.001**
GPT (U/L)	37.5 ± 82.5	46.3 ± 66.0	0.054	45.7 ± 46.1	38.5 ± 100.2	0.108	39.5 ± 40.6	45.9 ± 144.1	0.178
INR ratio	1.43 ± 1.25	1.43 ± 1.18	0.995	1.25 ± 0.49	1.55 ± 1.75	**<0.001**	1.34 ± 0.99	1.69 ± 2.10	**<0.001**
AAR ratio	1.39 ± 0.72	1.56 ± 0.74	**<0.001**	1.17 ± 0.51	1.58 ± 0.76	**<0.001**	1.32 ± 0.66	1.74 ± 0.76	**<0.001**
APRI score	0.59 ± 1.34	0.72 ± 0.77	0.073	0.57 ± 0.54	0.68 ± 1.55	0.105	0.55 ± 0.54	0.89 ± 2.24	**<0.001**
FIB-4 score	2.66 ± 3.00	3.31 ± 2.75	**<0.001**	1.86 ± 1.20	3.51 ± 3.66	**<0.001**	2.43 ± 2.22	4.24 ± 4.48	**<0.001**
BLR	0.02 ± 0.02	0.03 ± 0.06	**<0.001**	0.02 ± 0.02	0.02 ± 0.05	**<0.001**	0.02 ± 0.03	0.03 ± 0.04	**<0.001**
NLR	5.57 ± 5.27	10.78 ± 18.67	**<0.001**	5.18 ± 4.48	8.24 ± 14.32	**<0.001**	5.92 ± 10.64	11.43 ± 10.76	**<0.001**
PLR	220.1 ± 139.5	303.8 ± 315.0	**<0.001**	213.7 ± 126.5	264.6 ± 253.9	**<0.001**	230.8 ± 198.1	296.5 ± 225.0	**<0.001**
EBR	1.87 ± 4.31	1.06 ± 2.35	**<0.001**	1.61 ± 2.85	1.66 ± 4.58	0.800	1.89 ± 4.19	1.01 ± 2.47	**<0.001**
ELR	0.03 ± 0.10	0.02 ± 0.05	0.054	0.02 ± 0.04	0.03 ± 0.11	**0.024**	0.03 ± 0.09	0.02 ± 0.06	0.119
LMR	2.71 ± 2.07	2.33 ± 1.56	**<0.001**	2.82 ± 1.74	2.40 ± 1.70	**<0.001**	2.66 ± 1.83	2.43 ± 2.23	**0.040**

Values are expressed as means ± standard deviations. *p* values were calculated by Student’s *t*-tests. Bold numbers indicate *p* value lower than 0.05. F: female; M: male; CCI: Charlson Comorbidity Index; CRP: C-reactive protein; LDH: lactate dehydrogenase; GGT: gamma glutamyl transferase; GOT: glutamic oxaloacetic transaminase; GPT: glutamate pyruvate transaminase; INR: international normalized ratio; AAR: AST/ALT ratio; APRI: AST to Platelet Ratio Index; FIB-4: Fibrosis-4 index; BLR: basophil-to-lymphocyte ratio; NLR: neutrophil-to-lymphocyte ratio; PLR: platelet-to-lymphocyte ratio; EBR: eosinophil-to-basophil ratio; ELR: eosinophil-to-lymphocyte ratio; LMR: lymphocyte-to-monocyte ratio.

**Table 2:** Clinical and phenotypical characteristics of COVID-19 patients based on median values of inflammatory and liver features.

	CRP			Neutrophils			FIB-4		
Variable	<73.67 (*n* = 933)	≥73.67 (*n* = 934)	*p*	<4.89 (*n* = 976)	≥4.89 (*n* = 982)	*p*	<2.17 (*n* = 810)	≥2.17 (*n* = 818)	*p*
Age	65.1 ± 17.2	69.9 ± 14.5	**<0.001**	65.2 ± 16.0	69.8 ± 15.7	**<0.001**	61.0 ± 15.8	74.3 ± 13.0	**<0.001**
Sex (F/M)	425/508	315/618	**<0.001**	416/560	363/618	**0.003**	350/460	281/537	**<0.001**
CCI	2.86 ± 2.42	3.42 ± 2.33	**<0.001**	2.87 ± 2.34	3.43 ± 2.46	**<0.001**	2.36 ± 2.28	4.01 ± 2.29	**<0.001**
Oxygen saturation (SO_2_%)	94.4 ± 4.0	89.8 ± 4.4	**<0.001**	93.9 ± 4.5	90.3 ± 8.4	**<0.001**	93.1 ± 6.0	90.8 ± 8.0	**<0.001**
Leukocytes (×10^9^/L)	6.58 ± 3.46	8.66 ± 5.11	**<0.001**	5.07 ± 2.98	10.20 ± 4.27	**<0.001**	8.15 ± 3.99	7.18 ± 4.99	**<0.001**
Lymphocytes (×10^9^/L)	1.37 ± 1.61	0.99 ± 0.66	**<0.001**	1.18 ± 0.72	1.19 ± 1.62	0.172	1.28 ± 0.81	1.07 ± 1.70	**0.002**
Neutrophils (×10^9^/L)	4.57 ± 2.71	7.01 ± 4.18	**<0.001**	3.31 ± 0.95	8.30 ± 3.80	**<0.001**	6.17 ± 3.76	5.51 ± 3.55	**<0.001**
Platelets (×10^9^/L)	217.9 ± 94.9	231.7 ± 98.5	**0.002**	195.1 ± 80.1	254.2 ± 104.4	0.107	275.1 ± 102.8	177.0 ± 59.9	**<0.001**
Basophils (×10^9^/L)	0.021 ± 0.022	0.022 ± 0.020	0.527	0.017 ± 0.018	0.024 ± 0.021	0.475	0.025 ± 0.021	0.017 ± 0.016	**<0.001**
Eosinophils (×10^9^/L)	0.06 ± 0.28	0.03 ± 0.05	**0.001**	0.03 ± 0.07	0.04 ± 0.27	0.527	0.06 ± 0.30	0.02 ± 0.05	**<0.001**
Monocytes (×10^9^/L)	0.56 ± 0.31	0.60 ± 2.34	0.548	0.52 ± 2.31	0.63 ± 0.41	0.241	0.60 ± 0.32	0.56 ± 2.50	0.692
CRP (mg/L)	31.2 ± 21.4	173.6 ± 97.4	**<0.001**	62.9 ± 61.1	142.3 ± 115.1	**<0.001**	95.8 ± 103.3	113.4 ± 98.6	**<0.001**
D-dimer (μg/mL)	1.66 ± 4.82	3.04 ± 9.60	**0.001**	1.37 ± 4.45	3.35 ± 9.79	**<0.001**	2.00 ± 5.59	2.76 ± 8.95	0.065
Fibrinogen (mg/dL)	553.4 ± 122.4	812.3 ± 240.8	**<0.001**	627.9 ± 187.0	725.7 ± 255.1	0.763	687.2 ± 254.6	692.3 ± 221.7	0.903
Ferritin (ng/mL)	724 ± 632	2011 ± 2566	**<0.001**	1046 ± 1474	1791 ± 2437	0.226	1414 ± 2243	1474 ± 1872	0.883
Procalcitonin (ng/mL)	0.09 ± 0.04	0.77 ± 1.40	0.100	0.10 ± 0.06	0.92 ± 1.52	0.142	0.51 ± 1.35	0.74 ± 1.10	0.547
Glucose (mg/dL)	199.8 ± 82.6	199.6 ± 126.5	0.117	194.1 ± 83.3	182.1 ± 110.2	0.117	177.4 ± 128.6	201.1 ± 79.7	0.670
Cholesterol (mg/dL)	162.5 ± 23.3	125.2 ± 28.8	0.997	127.5 ± 72.8	137.9 ± 99.4	0.971	137.6 ± 33.6	134.5 ± 16.3	0.980
LDH (U/L)	521.2 ± 311.0	714.8 ± 362.8	**<0.001**	554.4 ± 299.9	682.8 ± 389.4	**<0.001**	536.4 ± 218.7	712.2 ± 445.2	**<0.001**
GGT (U/L)	49.9 ± 48.7	101.9 ± 173.5	**<0.001**	56.6 ± 59.4	97.5 ± 177.5	0.978	81.5 ± 102.5	69.1 ± 157.6	0.404
GOT (U/L)	38.8 ± 30.2	55.7 ± 134.3	**<0.001**	41.1 ± 29.5	53.7 ± 135.5	0.265	34.3 ± 23.6	61.2 ± 137.2	**<0.001**
GPT (U/L)	36.1 ± 40.7	44.8 ± 96.5	**0.020**	34.5 ± 33.0	46.4 ± 99.4	0.108	38.0 ± 42.4	43.2 ± 96.9	0.159
INR ratio	1.34 ± 1.04	1.48 ± 1.44	0.050	1.34 ± 0.97	1.50 ± 1.56	**<0.001**	1.36 ± 1.34	1.47 ± 1.31	0.144
AAR ratio	1.40 ± 1.32	1.50 ± 0.67	0.054	1.45 ± 0.70	1.44 ± 1.27	**<0.001**	1.14 ± 0.51	1.76 ± 1.30	**<0.001**
APRI score	0.54 ± 0.53	0.70 ± 1.50	**0.003**	0.63 ± 0.65	0.61 ± 1.47	0.105	0.34 ± 0.22	0.92 ± 1.55	**<0.001**
FIB-4 score	2.52 ± 2.24	3.12 ± 3.29	**<0.001**	3.01 ± 2.66	2.67 ± 3.15	**<0.001**	1.37 ± 0.48	4.30 ± 3.54	**<0.001**
Bas/Lym ratio	0.02 ± 0.03	0.03 ± 0.04	**<0.001**	0.01 ± 0.02	0.02 ± 0.04	**<0.001**	0.02 ± 0.04	0.02 ± 0.03	**0.027**
Neu/Lym ratio	4.52 ± 5.45	9.64 ± 14.02	**<0.001**	3.54 ± 2.62	10.56 ± 14.18	**<0.001**	6.97 ± 14.29	7.52 ± 7.77	0.337
Plat/Lym ratio	199.8 ± 141.9	295.8 ± 255.8	**<0.001**	199.1 ± 128.4	293.6 ± 258.3	**<0.001**	274.3 ± 264.9	231.4 ± 165.1	**<0.001**
Eos/Bas ratio	2.21 ± 4.86	1.20 ± 2.34	**<0.001**	1.88 ± 3.29	1.54 ± 4.24	0.800	2.13 ± 4.69	1.24 ± 2.90	**<0.001**
Eos/Lym ratio	0.04 ± 0.11	0.03 ± 0.05	**0.010**	0.02 ± 0.05	0.03 ± 0.10	**0.024**	0.041 ± 0.114	0.020 ± 0.051	**<0.001**
Lym/Mon ratio	2.82 ± 1.76	2.44 ± 2.04	**<0.001**	3.04 ± 1.78	2.18 ± 1.93	**<0.001**	2.60 ± 1.84	2.65 ± 2.07	0.557

Values are expressed as means ± standard deviations. *p* values were calculated by Student’s *t*-tests. Bold numbers indicate *p* value lower than 0.05. F: female; M: male; CCI: Charlson Comorbidity Index; CRP: C-reactive protein; LDH: lactate dehydrogenase; GGT: gamma glutamyl transferase; GOT: glutamic oxaloacetic transaminase; GPT: glutamate pyruvate transaminase; INR: international normalized ratio; AAR: AST/ALT ratio; APRI: AST to Platelet Ratio Index; FIB-4: Fibrosis-4 index; BLR: basophil-to-lymphocyte ratio; NLR: neutrophil-to-lymphocyte ratio; PLR: platelet-to-lymphocyte ratio; EBR: eosinophil-to-basophil ratio; ELR: eosinophil-to-lymphocyte ratio; LMR: lymphocyte-to-monocyte ratio.

**Table 3:** (**a**) Multiple logistic regression model using clinical, inflammatory and liver markers as important predictors of mortality plus ICU in COVID-19 patients: interaction between CCI and CRP. (**b**) Multiple logistic regression model using clinical, inflammatory and liver markers as important predictors of mortality plus ICU in COVID-19 patients: interaction between FIB-4 and CCI. (**c**) Multiple logistic regression model using clinical, inflammatory and liver markers as important predictors of mortality plus ICU in COVID-19 patients: interaction between FIB-4 and CRP. (**d**) Multiple logistic regression model using clinical, inflammatory and liver markers as important predictors of mortality plus ICU in COVID-19 patients: interaction between FIB-4 and NEU.

(a)		
Variable	β Coefficients (CI 95%)	*p*
Age (years)	0.0653 (0.0464, 0.0842)	**<0.001**
Sex (Female)	−0.5413 (−1.0090, −0.0735)	**0.023**
Neutrophils (×10^9^/L)	0.0972 (0.0370, 0.1574)	**0.002**
D-dimer (μg/mL)	0.0218 (−0.0037, 0.0472)	0.093
Oxygen saturation (SO_2_ < 92%)	0.6359 (0.1909, 1.0809)	**0.005**
FIB-4	0.2080 (0.1046, 0.3113)	**<0.001**
CCI * CRP	0.0013 (0.0007, 0.0018)	**<0.001**
R^2^	0.3093	**<0.001**
(**b**)		
**Variable**	**β coefficients (CI 95%)**	***p***
Age (years)	0.0776 (0.0598, 0.0955)	**<0.001**
Sex (Female)	−0.6635 (−1.1213, −0.2056)	**0.005**
Neutrophils (×10^9^/L)	0.0595 (−0.0026, 0.1216)	0.060
D-dimer (μg/mL)	0.0249 (−0.0009, 0.0507)	0.059
Oxygen saturation (SO_2_ < 92%)	0.6374 (0.1976, 1.0772)	**0.005**
CRP (mg/L)	0.0039 (0.0017, 0.0061)	**<0.001**
FIB-4 * CCI (no age)	0.0307 (0.0137, 0.0477)	**<0.001**
R^2^	0.2838	**<0.001**
(**c**)		
Age (years)	0.0655 (0.0471, 0.0839)	**<0.001**
Sex (Female)	−0.5982 (−1.0728, −0.1236)	**0.013**
Neutrophils (×10^9^/L)	0.0763 (0.0205, 0.1321)	**0.007**
D-dimer (μg/mL)	0.0227 (−0.0027, 0.0482)	0.080
Oxygen saturation (SO_2_ < 92%)	0.5183 (0.0658, 0.9709)	**0.025**
CCI (no age)	0.2094 (0.0962, 0.3226)	**<0.001**
FIB-4 * CRP	0.0014 (0.0009, 0.0020)	**<0.001**
R^2^	0.3134	**<0.001**
(**d**)		
Age (years)	0.0601 (0.0408, 0.0793)	**<0.001**
Sex (Female)	−0.5279 (−1.0003, -0.0555)	**0.028**
CRP (mg/L)	0.0032 (0.0012, 0.0053)	**0.002**
D-dimer (μg/mL)	0.0225 (−0.0027, 0.0477)	0.080
Oxygen saturation (SO_2_ < 92%)	0.5081 (0.0519, 0.9642)	**0.029**
CCI (no age)	0.2202 (0.1077, 0.3327)	**<0.001**
FIB-4 * NEU	0.0383 (0.0210, 0.0556)	**<0.001**
R^2^	0.3151	**<0.001**

Bold numbers indicate *p* < 0.05.

## Data Availability

The data presented in this study are available on request from the corresponding author.
